# Investigation of the Effect of Therapeutic Plasma Exchange for TAFRO Syndrome: A Pilot Study

**DOI:** 10.3390/biomedicines12040849

**Published:** 2024-04-11

**Authors:** Kosuke Sonoda, Masamichi Komatsu, Yoko Ozawa, Hiroshi Yamamoto, Yuji Kamijo

**Affiliations:** 1 Department of Nephrology, Shinshu University Hospital, Matsumoto 390-8621, Japan; kosuke_s@live.jp (K.S.); yujibeat@shinshu-u.ac.jp (Y.K.); 2 First Department of Internal Medicine, Shinshu University School of Medicine, Matsumoto 390-8621, Japan; ozyoko@shinshu-u.ac.jp (Y.O.); yama5252@shinshu-u.ac.jp (H.Y.); 3 Department of Respiratory Medicine, Iida Municipal Hospital, Iida 395-8502, Japan

**Keywords:** TAFRO syndrome, therapeutic plasma exchange, thrombotic microangiopathy

## Abstract

TAFRO syndrome is a rare systemic inflammatory disorder with a fatal course. Nevertheless, a definitive treatment strategy has not yet been established. Anti-inflammatory therapies, including glucocorticoid treatment and immunosuppressants, have not been satisfactory. Therefore, new treatment options are needed for patients with TAFRO syndrome. The effectiveness of therapeutic plasma exchange (TPE) has mainly been reported in several case reports. In this case series study, we investigated the effect of TPE on TAFRO syndrome. We reviewed six consecutive cases with TAFRO syndrome treated at Shinshu University Hospital. All of them underwent TPE. A significant improvement in mean blood pressure, albumin, total bilirubin, and C-reactive protein was observed after TPE. Furthermore, early TPE treatment was suggested to have an impact on the prognosis. More intensive studies are needed to emphasize the overall conclusion obtained that TPE can be an effective/acceptable treatment option for TAFRO syndrome.

## 1. Introduction

TAFRO syndrome is a systemic inflammatory disorder characterized by thrombocytopenia (T), anasarca (A), fever (F), reticulin myelofibrosis and/or renal dysfunction (R), and organomegaly (O) [1]. The histopathological features of lymph nodes in TAFRO syndrome resemble those of Castleman’s disease [2]. Therefore, TAFRO syndrome is considered a subtype of idiopathic multicentric Castleman disease [3]. However, the clinical course of TAFRO syndrome is more aggressive than that of Castleman disease, and patients often have a poor prognosis despite multidisciplinary treatment. Diagnostic criteria and disease severity classifications for TAFRO syndrome have been proposed for early diagnosis and treatment [4,5].

There is no definitive treatment strategy for TAFRO syndrome owing to its rarity and unknown etiology. In general, glucocorticoids (GCs) with or without anti-interleukin-6 monoclonal antibody siltuximab (or tocilizumab, if siltuximab is not available) are the first-line treatment. In the case of inadequate response to the first-line treatment, additional treatment using rituximab (anti-CD20 antibody), cyclosporin A (CsA), immunomodulatory agent, combination chemotherapy, etc., is considered [4,6]. In clinical practice, a combination of these treatments is often used.

In addition to pharmacotherapy, there have been case reports of patients undergoing therapeutic plasma exchange (TPE) for TAFRO syndrome, though its clinical effects remain obscure. We investigated the effectiveness of TPE in TAFRO syndrome patients.

## 2. Materials and Methods

### 2.1. Study Designs and Participants

This was a retrospective, single-center study. We reviewed the medical records of consecutive patients with TAFRO syndrome who were treated in our institution from January 2010 to December 2023.

This study was approved by the Ethics Committee of the Shinshu University School of Medicine (Approval number 6048) and was performed following the Declaration of Helsinki and its subsequent amendments. The Institutional Review Board of Shinshu University School of Medicine waived the need for informed consent owing to its retrospective design.

### 2.2. Clinical Findings and Treatment

We investigated baseline clinical and physiological data and treatment strategies for TAFRO syndrome such as age, gender, date of event, outcome, body weight, blood pressure, laboratory data (hemoglobin, hematocrit, platelet count, creatinine, total bilirubin (T.Bil), C-reactive protein (CRP)), Sequential Organ Failure Assessment (SOFA) score, and pharmacotherapy, which were obtained from medical records. The severity of TAFRO syndrome was assessed according to the Castleman Disease Collaborative Network severity classification [6]. Outcome was assessed as survival to hospital discharge or death. In cases of death, the cause of death was also assessed. We also obtained data on apheresis conditions, including types and volume of replacement fluid and the number of TPE sessions. The estimated plasma volume (PV), which is the reference for the volume of replacement fluid, was calculated by the following formula: estimated PV = (0.065 × weight) × (1 − hematocrit) [7]. Furthermore, to detect the clinical effect of TPE, mean blood pressure (mBP); serum levels of albumin, creatinine, T.Bil, and CRP; and platelet count were evaluated before and after TPE. mBP data immediately before and after TPE were obtained. Other data were obtained using blood samples taken on the morning of the TPE performed day and those taken the next morning. Moreover, we investigated the timing of the initiation of TPE and pharmacotherapy.

### 2.3. Statistical Analysis

Continuous data were presented as medians (interquartile range) and categorical data as the number of cases in each group. The paired Wilcoxon-rank test was used to compare clinical and serological data before and after TPE. We performed the analysis for each TPE session and also analyzed the first session of each case as a sensitivity analysis. The statistical analyses were performed using StatFlex^®^ Version 7.0 (Artech, Osaka, Japan). The statistical significance was established at *p*-values of <0.05.

## 3. Results

### 3.1. Patient Characteristics

There were six patients with TAFRO syndrome, and all of them were classified as severe upon hospital admission. They showed a SOFA score of 1 to 7 upon hospital admission, which increased to 7 to 16 upon TPE initiation. Cases 1, 5, and 6 required tracheal intubation during hospitalization. All patients underwent TPE, which was performed using the PLASAUT EZ (Asahikasei Medical Co., Ltd., Tokyo, Japan) or ACH-Σ (PLASAUTO Σ in overseas models) (Asahikasei Medical Co., Ltd., Tokyo, Japan) blood purification systems with the membrane plasma separation method using Plasmaflo OP (Asahikasei Medical Co., Ltd., Tokyo, Japan). In all TPE sessions, Nafamostat mesylate was used as the anti-coagulant, and non-tunneled central venous catheters were installed during hemodialysis therapy for vascular access. The clinical course of two of the six patients was previously reported (Cases 1, 2) [8]. For clinical information, details of the pharmacotherapy and TPE are summarized in Table 1. Replacement fluid equivalent to 0.7 to 1.5 times the patient’s estimated plasma volume was used for TPE. Cases 1, 2, 3, and 6 used fresh frozen plasma (FFP) as a replacement fluid throughout their clinical course. The type of replacement fluid in cases 4 and 5 was an albumin solution for the first TPE sessions, but thereafter, FFP alone or a mixture of albumin solution and FFP were used in these cases. All cases underwent TPE multiple times. In case 1, TPE was performed 35 times, while in the other cases, it was 2 to 8 times. The frequency of TPE was basically daily or every other day, but this rate was adjusted according to the clinical condition of the patient. Days from onset to TPE initiation varied from 18 to 53 days. Two patients died of multiple organ failure and infection; however, four patients survived.

### 3.2. Changes in Clinical Data before and after TPE

**Figure 1:**
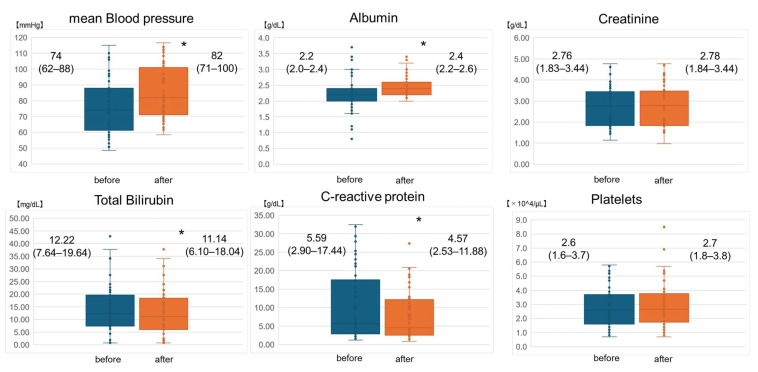
Changes in clinical data before and after therapeutic plasma exchange. Data have been presented as median values (interquartile range). * *p* < 0.05.

In this study, a total of 56 TPE sessions were performed for the six patients. Changes in clinical data before and after TPE are shown in Figure 1. The median (interquartile range) mBP values before and after TPE were 74 (62–88) and 82 (71–100) mmHg, respectively (*p* < 0.01). The median serum albumin levels before and after TPE were 2.2 (2.0–2.4) and 2.4 (2.2–2.6) g/dL, respectively (*p* < 0.01). The median serum creatinine levels before and after TPE were 2.76 (1.83–3.44) and 2.78 (1.84–3.46) mg/dL, respectively (*p* = 0.365). The median T.Bil levels before and after TPE were 12.22 (7.64–19.64) and 11.14 (6.10–18.04) mg/dL, respectively (*p* < 0.01). The median CRP levels before and after TPE were 5.59 (2.90–17.44) and 4.57 (2.53–11.88) mg/dL, respectively (*p* < 0.01). The median platelet counts before and after TPE were 2.6 (1.6–3.7) and 2.7 (1.8–3.8) × 10^4^/μL, respectively (*p* = 0.338). Before and after TPE, mBP and serum albumin levels rose, and serum T.Bil and CRP levels were significantly improved after TPE. Regarding serum creatinine levels and platelets, no fixed trend was observed. Changes in clinical data for each TPE session are shown in Figure S1 in the Supplementary File.

**Figure 2:**
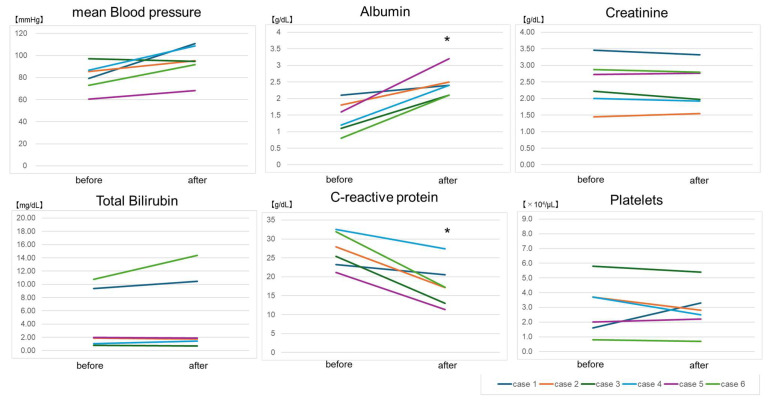
Changes in clinical data before and after therapeutic plasma exchange on the first session. * *p* < 0.05.

In addition, the analysis of the first session of each case showed the same trend and statistically significant differences, especially in serum albumin level and CRP (*p* = 0.031, *p* = 0.031, respectively) (Figure 2). Changes in clinical data may be affected not only by the effects of TPE but also by the effects of other concurrent treatments, such as anti-inflammatory therapy, blood transfusions, and hemodialysis. Concerning adverse events, chest pain and abdominal pain occurred in one patient each after TPE, and these events quickly improved. The causal relationship between these events and TPE was unknown.

### 3.3. Timing of Initiation of TPE and Pharmacotherapy

In all cases, GC treatment was started before TPE. Immunosuppressants, such as CsA, tocilizumab, and rituximab, were administrated after TPE in cases 3, 4, and 5, and these patients survived. On the other hand, in case 6, GC and tocilizumab were administrated before TPE, and the patient died. The timing of initiation of TPE and pharmacotherapy can affect the prognosis. In addition, the period from symptom onset to TPE initiation tended to be longer than 1 month in fatal cases (Case 1, 6), suggesting the importance of the early initiation of TPE.

## 4. Discussion

We summarized the cases of patients with TAFRO syndrome in our hospital. There is no definitive treatment strategy for TAFRO syndrome, owing to its rarity. In particular, the usefulness of TPE for TAFRO syndrome has not been established. However, TPE was performed in all cases at our hospital, suggesting that pharmacotherapy alone is often unable to control the disease activity of TAFRO syndrome. To investigate the effects of TPE on TAFRO syndrome, we summarized the past 10 case reports of patients who underwent TPE for TAFRO syndrome in Table 2 [8,9,10,11,12,13,14,15,16]. Similarly, in our case series, TPE was not performed alone but in combination with various drug treatments in all cases. Meguri et al. [13] reported that TPE was effective in reducing jaundice and kidney dysfunction caused by multiorgan failure. Otsuka et al. [15] reported that TPE effectively improved inflammatory reactions and anasarca. In summary, TPE was effective in five cases and ineffective in the remaining cases. No adverse events related to TPE were reported.

It has been hypothesized that the pathogenesis of TAFRO syndrome involves an increase in inflammatory cytokines, such as IL-6 and vascular endothelial growth factor (VEGF). The primary purpose of TAFRO syndrome treatment is to control systemic inflammation. Various immunosuppressive treatments, including GC, have been used to treat TAFRO syndrome.

TPE has been used to remove pathogenic substances such as cytokines, antigen–antibody immunocomplexes, autoantibodies, abnormal immunoglobulin, albumin-binding toxic substances, and bilirubin from the plasma of patients and supply deficient plasma components [17]. The effectiveness of TPE for the treatment of TAFRO syndrome was hypothesized as follows.

First, TPE is able to remove inflammatory cytokines immediately. In the current study, improvements in CRP were seen after TPE, regardless of the small number of investigations. This might be due to the quick removal of inflammatory cytokines. TPE can exert a bridging effect until the anti-inflammatory and immunosuppressive treatments become effective. The effects of immunosuppressants take several weeks to manifest [18]. Therefore, it would be important to quickly remove the inflammatory cytokines from the patient’s plasma by TPE until the immunosuppressive therapy becomes effective. For patients with acute antibody-mediated rejection after kidney transplantation, it has been reported that the addition of rituximab to TPE exerts a higher improvement effect than TPE alone [19]. In this combination therapy via TPE and pharmacotherapy, the order of TPE and drug administration may be crucial for sufficient efficacy of the immunosuppressive therapy. We should consider that TPE can remove not only inflammatory substances but also important immunosuppressive medicines such as rituximab. Otsuka et al. [15] reported the efficacy of additional immunosuppressant administration after TPE. When TPE is performed after starting immunosuppressive therapy, additional therapy to replace the immunosuppressants removed by TPE may be necessary. In our case series, the patient who received immunosuppressants after TPE survived, suggesting the importance of additional immunosuppressive therapy after TPE. Furthermore, immediate elimination of inflammatory cytokines in TAFRO syndrome patients may attenuate their disease activity and can reduce the need for long-term, continuous strong anti-inflammatory immunosuppressive treatments including GC and immunosuppressants. Since the strong immunosuppressive therapy used for TAFRO syndrome appeared to increase infections and lead to fatal outcomes [20], TPE may prevent them. Currently, the appropriate timing to perform TPE for TAFRO syndrome is unknown. In this study, treatment with TPE was mainly selected for cases judged to be GC-resistant after GC administration. There are limited case reports describing TPE for TAFRO syndrome in detail, making it difficult to discuss this issue. However, we think an early introduction of TPE would be reasonable in TAFRO cases with inadequate or failed responses to the current therapy. In the current study, the patients who underwent TPE in a relatively early phase tended to survive, suggesting the importance of the early introduction of TPE.

Secondly, TPE could improve systemic and circulatory dysfunction by replacing the deficient essential plasma components, including albumin, coagulation factors, and immunoglobulin. TAFRO syndrome causes severe cytokine storm; moreover, the combination of kidney dysfunction, endothelial cell death, and acute-phase hypoalbuminemia can lead to capillary leak syndrome and anasarca in severe cases [21]. TPE can replenish cytokine-storm-associated loss factors including albumin and protective plasma proteins which are important for coagulation and fibrinolysis, and it counteracts inflammation and vascular leakage. In the current study, an albumin solution or FFP was used, and serum albumin levels tended to increase after TPE, suggesting the albumin replacement effects of TPE. Furthermore, mBP tended to increase after TPE, suggesting an improvement in circulatory dysfunction. Past studies reported a decrease in ADAMTS13 activity, which is crucial for fibrinolysis, in three cases with TAFRO syndrome [11,12,14]. TPE can also replenish ADAMTS13, which may improve disease status in these patients. In the current study, serum ADAMTS13 activity was evaluated in two of six cases before TPE, but no decrease was observed in any of them.

Third, TPE may be effective in treating thrombotic microangiopathy (TMA). Kidney dysfunction and thrombocytopenia are frequently observed in TAFRO syndrome [22]. Although the pathogenesis of the kidneys’ involvement in TAFRO syndrome is not clearly understood, cytokine storms involving IL-6 and VEGF have been suggested to cause immunological disorders and vascular endothelial cell damage. In kidney histopathological examination, TMA-like lesions caused by glomerular endothelial cell damage have been reported in TAFRO syndrome patients [22,23,24]. Although TMA is caused by a variety of etiologies, TPE is commonly introduced when a patient is diagnosed with TMA, often before the underlying etiology is identified. This requirement is appropriate for many clinical settings [24]. TPE may also be effective in TMA associated with TAFRO syndrome. In the current study, there were no cases in which a kidney biopsy was performed, and the relationship between TPE’s effectiveness and renal TMA-like lesions was unclear. Noda et al. reported that TPE may be ineffective even when TMA-like lesions are observed in kidney tissues [11]; therefore, further investigations are required.

TPE should be performed based on the understanding of the indications [25]; however, the indication for TPE for TAFRO syndrome is not clearly mentioned in the guidelines [6,26]. However, the American Society for Apheresis (ASFA) guidelines state that TPE treatment may be considered in hemophagocytic lymphohistiocytosis and sepsis with multiorgan failure, which are similar to TAFRO syndrome in that cytokine storm is the main clinical manifestation and the target of TPE treatment. Therefore, it is speculated that TPE would also be reasonable for consideration in TAFRO syndrome, which belongs to a group of diseases associated with cytokine storms.

The appropriate apheresis technique and prescription of TPE for TAFRO syndrome is unknown; therefore, it should be performed considering the pathophysiology and the hypothesized mechanism of effectiveness. In the current study, the plasma separation method and anti-coagulants were decided with reference to the commonly used TPE method for critically ill patients in Japan. In the case of TAFRO syndrome, the replacement solution should be selected depending on the expected TPE effects. Compared to TPE using FFP, TPE using an albumin solution has the advantage of having a lower risk of causing allergic reactions and unknown virus infections, in addition to its lower medical costs; therefore, an albumin solution is recommended as a replacement fluid for TPE generally. However, albumin solutions do not contain important plasma components including coagulation factors and immunoglobulins; TPE using an albumin solution would not replenish these components, which makes it difficult to recommend albumin solutions in cases with hemorrhagic disease and infection. In the current study, only 2 sessions among a total of 56 TPE sessions selected albumin solution alone as the replacement solution. TAFRO syndrome is prone to bleeding due to low platelet counts and coagulation factor deficiency, and immunoglobulin deficiency and infections are more likely to occur due to immunosuppressive therapy and poor general condition. Therefore, we selected FFP as a replacement fluid for TPE. Generally, the plasma exchange volume of a single TPE session is determined with reference to the estimated PV calculated by a specific formula, e.g., estimated PV = (0.065 × weight) × (1 − hematocrit). Typically, 1 to 1.5 PV was exchanged in a single session. In this study, the amount of replacement fluid was relatively low, especially in fatal cases (Case 1, 6). This result would be influenced by the increase in the estimated PV due to body weight gain associated with anasarca and hematocrit decrease associated with persistent inflammation, and a lower replacement fluid volume would not be recommended at least.

The current study has several limitations. First, our treatment strategy does not fully follow the international, evidence-based consensus treatment guidelines [6]. This may have influenced the poor outcomes. Among fatal cases, case 1 happened before the guidelines’ publication, and the treatment of case 6 was generally in line with the guidelines. Second, the number of TPEs in case 1 appear to be higher than the others, which may have added bias to the study results. A sensitivity analysis was performed for the first TPE session to confirm the robustness of the results. Third, this study is a small retrospective study at a single center. However, this is a reasonable study design because TAFRO syndrome is rare, and we need to evaluate the details of TPE.

## 5. Conclusions

Establishing a standard treatment strategy for TAFRO syndrome is challenging because of its rarity and heterogeneity. TPE is currently performed on an exploratory basis, as other treatment options are lacking.

This study indicated the possibility that a combination treatment including GC, immunosuppressants, and TPE may improve the inflammatory response, and suggested that the early initiation of TPE may be effective. Adverse events were not serious. More intensive studies are needed to emphasize the overall conclusion obtained that TPE can be an effective/acceptable treatment option for TAFRO syndrome.

## Figures and Tables

**Table 1:** Summary of cases and details of therapeutic plasma exchange for TAFRO syndrome.

Case	Age, Gender	Pharmacotherapy	SOFA Score on Hospital Admission	SOFA Score on TPE Initiation	Days from Onset to TPE Initiation	Type and Volume of Replacement Fluid for the First TPE	Total Number of TPE Procedures	Outcome
1	43, male	GC	5	13	41	FFP, 0.76 PV	35	Died (multiple organ failure)
2	46, male	GC	3	7	18	FFP, 1.49 PV	8	Survived
3	74, male	GC, CsA	3	8	28	FFP, 1.31 PV	4	Survived
4	46, female	GC, TCZ, CsA, RTX	7	7	20	Alb, 1.17 PV	2	Survived
5	72, female	GC, TCZ	1	11	22	Alb, 1.25 PV	2	Survived
6	60, female	GC, TCZ	2	16	53	FFP, 1.16 PV	5	Died (infection)

Alb, albumin solution; CsA, cyclosporine A; FFP, fresh frozen plasma; GC, glucocorticoids; PV, patient plasma volume; RTX, rituximab; SOFA, Sequential Organ Failure Assessment; TCZ, tocilizumab; TPE, therapeutic plasma exchange.

**Table 2:** Summary of case reports on patients who underwent therapeutic plasma exchange for TAFRO syndrome.

	Age, Gender	Treatment	Response to TPE	Outcome
Yasuda, 2016 [9]	21, female	GC, CsA, Tac, R-CHOP,R-CEPP	Not effective	Survived
Hiramatsu, 2016 [10]	48, male	GC, TCZ, RTX, IVIG	Not effective	Survived
Ozawa, 2017 [8]	46, male	GC	Effective	Survived
Ozawa, 2017 [8]	43, male	GC	Effective	Died (multiple organ failure)
Noda, 2018 [11]	79, female	GC, RTX	Not effective	Survived
Mizuno, 2018 [12]	84, male	GC, TCZ	Not effective	Died (Aspiration pneumonia)
Meguri, 2019 [13]	52, female	GC, CY, TCZ, RTX	Effective	Survived
Nagai, 2021 [14]	57, male	GC, Tac, MMF	Not effective	Died
Otsuka, 2021 [15]	69, female	GC, TCZ, RTX	Effective	Survived
Sakaki, 2022 [16]	51, female	GC, CsA, RTX	Effective	Survived

CsA, cyclosporine A; CY, cyclophosphamide; GC, glucocorticoids; IVIG, intravenous immunoglobulin; MMF, mycophenolate mofetil; R-CEPP, rituximab/cyclophosphamide/etoposide/procarbazine/prednisolone; R-CHOP, rituximab/cyclophosphamide/adriamycin/vincristine/prednisolone; RTX, rituximab; Tac, tacrolimus; TCZ, tocilizumab; TPE, therapeutic plasma exchange.

## Data Availability

The datasets generated and/or analysed during the current study are not publicly available but are available from the corresponding author upon reasonable request.

## Supplementary Materials

The following supporting information can be downloaded at: https://www.mdpi.com/article/10.3390/biomedicines12040849/s1, Figure S1: Change of clinical data before and after therapeutic plasma exchange procedures.

## Abbreviations

Alb, albumin solution; ASFA, American Society for Apheresis; CRP, C-reactive protein; CsA, cyclosporine A; CY, cyclophosphamide; FFP, fresh frozen plasma; GC, glucocorticoids; IVIG, intravenous immunoglobulin; mBP, mean blood pressure; MMF, mycophenolate mofetil; R-CEPP, rituximab/cyclophosphamide/etoposide/pro-carbazine/prednisolone; R-CHOP, rituximab/cyclophosphamide/adriamycin/vincristine/pred-nisolone; RTX, rituximab; SOFA, Sequential Organ Failure Assessment; T.bil, total bilirubin; Tac, tacrolimus; TCZ, tocilizumab; TPE, therapeutic plasma exchange; VEGF, vascular endothelial growth factor.
