# Supplementary figures and images for: Investigation of the Effect of Therapeutic Plasma Exchange for TAFRO Syndrome: A Pilot Study
Source: Biomedicines. 2024 Apr 11;12(4):849. doi: 10.3390/biomedicines12040849 (PMC11048432; doi:10.3390/biomedicines12040849)

**Figure S1. Change of clinical data before and after therapeutic plasma exchange procedures.**

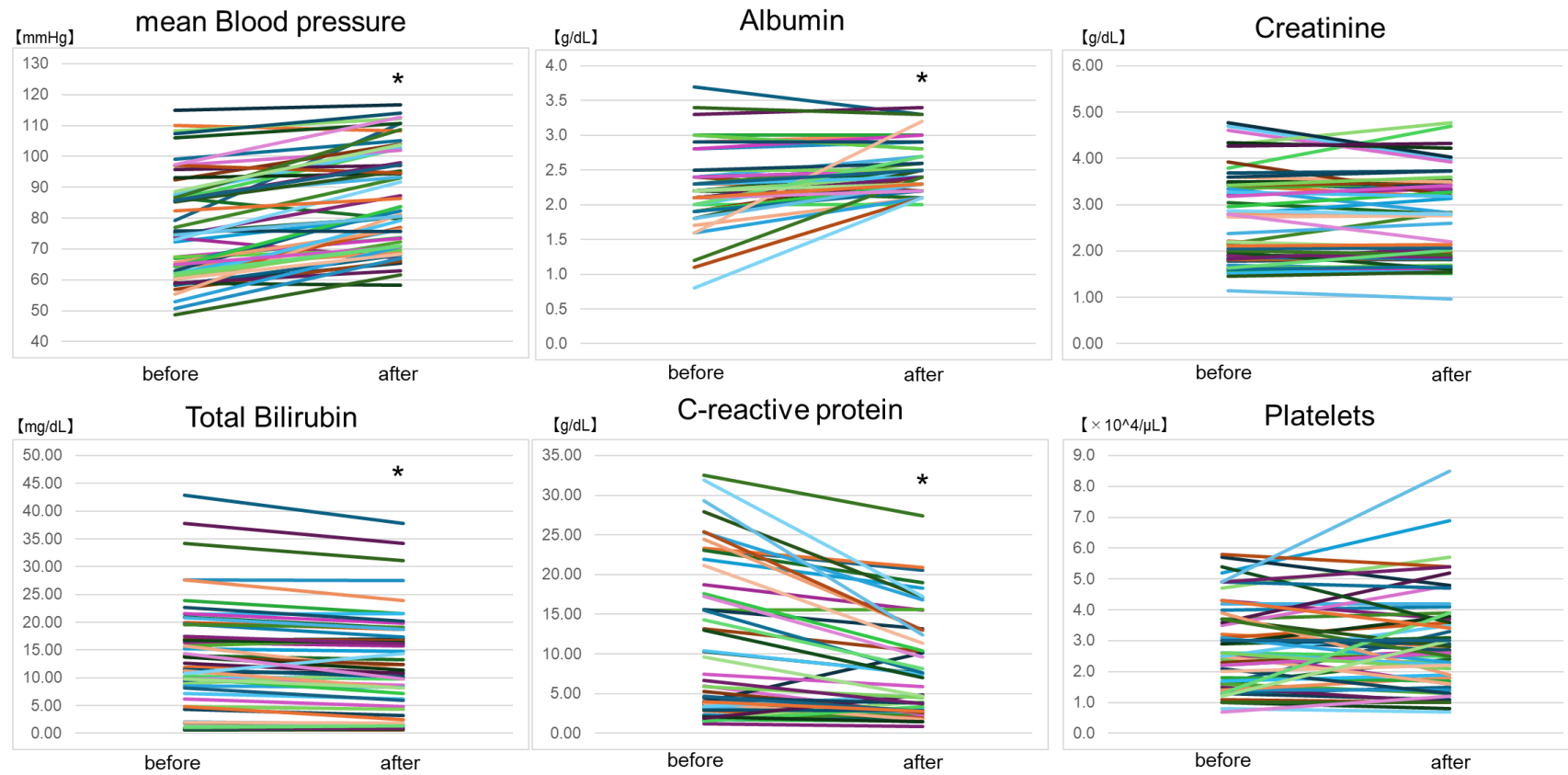

**\*  $p < 0.05$ .**

Supplement: Supplementary file 1 [file biomedicines-12-00849-s001.zip › biomedicines-2956810-supplementary.pdf]
